# A Combined *In Vitro/In Silico* Approach to Identifying Off-Target Receptor Toxicity

**DOI:** 10.1016/j.isci.2018.05.012

**Published:** 2018-05-18

**Authors:** Joseph Leedale, Kieran J. Sharkey, Helen E. Colley, Áine M. Norton, David Peeney, Chantelle L. Mason, Jean G. Sathish, Craig Murdoch, Parveen Sharma, Steven D. Webb

**Affiliations:** 1 EPSRC Liverpool Centre for Mathematics in Healthcare, Department of Mathematical Sciences, University of Liverpool, Liverpool L69 7ZL, UK; 2 School of Clinical Dentistry, University of Sheffield, Sheffield S10 2TA, UK; 3 MRC Centre for Drug Safety Science, Department of Molecular and Clinical Pharmacology, University of Liverpool, Liverpool L69 3GE, UK; 4 Department of Applied Mathematics, Liverpool John Moores University, Liverpool L3 3AF, UK; 5 Immuno and Molecular Toxicology, Drug Safety Evaluation, Bristol-Myers Squibb, 1 Squibb Drive, New Brunswick, NJ 08903, USA

**Keywords:** Toxicology, Computational Toxicology, Systems Biology

## Abstract

Many xenobiotics can bind to off-target receptors and cause toxicity via the dysregulation of downstream transcription factors. Identification of subsequent off-target toxicity in these chemicals has often required extensive chemical testing in animal models. An alternative, integrated *in vitro/in silico* approach for predicting toxic off-target functional responses is presented to refine *in vitro* receptor identification and reduce the burden on *in vivo* testing. As part of the methodology, mathematical modeling is used to mechanistically describe processes that regulate transcriptional activity following receptor-ligand binding informed by transcription factor signaling assays. Critical reactions in the signaling cascade are identified to highlight potential perturbation points in the biochemical network that can guide and optimize additional *in vitro* testing. A physiologically based pharmacokinetic model provides information on the timing and localization of different levels of receptor activation informing whole-body toxic potential resulting from off-target binding.

## Highlights

• Development of *in vitro/in silico* framework for identifying off-target toxicity
• Mathematical modeling of receptor signaling and related transcriptional activity
• Identification of key events in the signaling pathway
• Off-target receptor activation *in vivo* simulated using PBPK modeling

## Graphical Abstract

**Figure:**
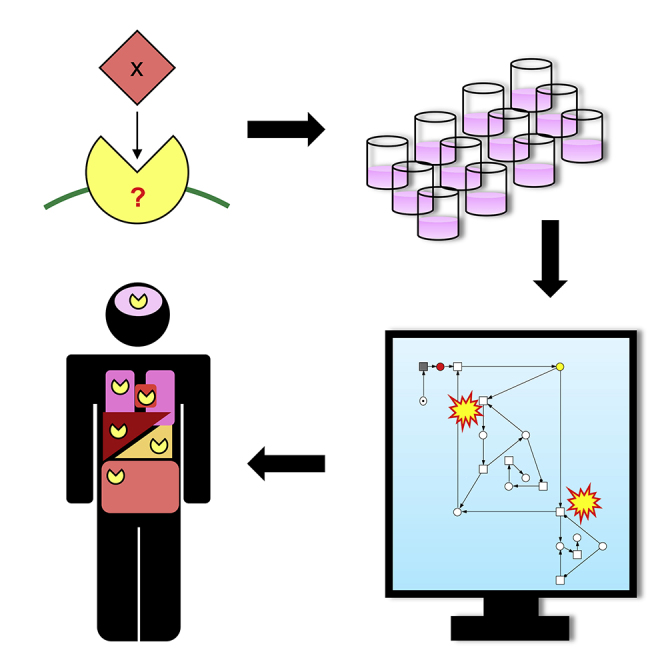

## Introduction

Many drugs are designed to interact specifically with cell surface, cytoplasmic, or nuclear receptors to produce a beneficial therapeutic effect. However, drugs can often bind to and interact with receptors that are not their intended targets, and such “off-target” binding may cause what is now often termed a molecular initiating event (MIE), e.g., receptor activation of toxicological relevance that may ultimately lead to an adverse drug reaction (ADR) (Edwards and Aronson, 2000, Guengerich, 2011, Muller and Milton, 2012). In many instances, ADRs can lead to significant morbidity and mortality as well as contribute to high levels of attrition during drug development (Lazarou et al., 1998, Pirmohamed et al., 2004). This can primarily be attributed to an incomplete understanding of the molecular mechanism of action of a given compound and the lack of ability to predict which receptors may be activated unintentionally.

The sole use of *in vitro*-based experimental strategies in the early stages of drug development and chemical testing is important but can lead to an unreliable and incomplete understanding of reactions (Coleman, 2011). Therefore, often considerable numbers of animals are used to screen out chemicals that may cause off-target toxicity, with figures for the UK reporting that 306,000 *in vivo* toxicology safety procedures were performed in 2014 (Home Office, 2015). In addition, the chemical industry used almost 345,000 animals in the EU for toxicological or other safety evaluations (European Commission, 2013), and in the United States 3–6 million fish are used annually for whole effluent toxicity testing (Scholz et al., 2013). Furthermore, pharmacokinetics and pharmacodynamics are significantly different between animal models and humans, diminishing their effectiveness in detecting toxicity through pre-clinical studies (Lauschke et al., 2016). There is therefore a clear need to develop scientific approaches to identify toxicologically relevant off-target receptor binding to reduce the burden of animal use in toxicity testing. The development of a more ethical, non-animal toolkit for initial chemical toxicological assessment using an integrated human-based *in vitro/in silico* system would enhance current strategies and may even expedite the drug development pipeline.

In intracellular signaling, ligand/receptor interactions lead to the activation of a distinct set of transcription factors, the effects of which tend to be tissue specific. Several companies now offer transcription factor activation profiling platforms, and so it is possible to identify and catalog the transcription factor activation profiles of toxicologically relevant receptors upon binding of their known ligands/drugs. It is assumed that transcription factor profiles generated from off-target receptor activation of any given drug can be matched against known ligand/receptor transcription profiles to predict which specific receptor (or class of receptors) has been activated in the initial off-target MIE. However, when testing off-target profiles of new compounds, the resulting transcription profile may not precisely match that of a known receptor (e.g., partial agonism or the binding of multiple receptors), and therefore a method of refinement is required to narrow the subset of off-target receptors. Our approach aims to refine the *in vitro* receptor identification process for off-target receptors by using information about the changes in receptor-mediated transcription factor activity following the introduction of a given compound and integrating this information with predictive *in silico* models and analysis. This approach allows for the identification of relevant perturbations in the transcription factor signaling pathway that signify the binding of a receptor or smaller range of receptors as well as other points of interest in the transcription factor signaling network that can contribute toward and guide subsequent off-target receptor identification.

Translating the wealth of knowledge on network interactions of cellular components to dynamic models is generally limited by the amount of available quantitative information to accompany these relationships, such as molecular amounts and reaction rates. However, qualitative dynamic network modeling can be used to compare with routinely generated semi-quantitative experimental time course data, where perturbations can provide valuable information about the system. *In silico* modeling of this type then provides a platform for the refinement of more quantitative (parameter-based) modeling (Fisher et al., 2013). In such a scenario, the network modeling method of Petri nets provides an effective tool, particularly in the complex, stochastic framework of molecular biological pathways (Chaouiya, 2007, Heiner et al., 2008, Heidary et al., 2015). Petri nets are often used to model multiple species and reactions without defining large quantities of unknown parameters, as modeling emphasis is on network topology and relative amounts of species rather than on specific reaction rates. This emphasis on network structure can then be translated to methods such as flux balance analysis and metabolic control analysis (MCA) without knowledge of rate constants, as was shown for the switching of the metabolic pathway in *E. coli* (Edwards et al., 2001, Kitano, 2002).

The identification of off-target receptor binding alone for a given compound is insufficient to predict significant off-target toxicity, and so we aim to provide additional information to support and refine the subsequent evaluation of toxic potential. This is achieved by translating knowledge of receptor binding properties and relative distribution of the receptor throughout the body to a whole-body response to the xenobiotic. This approach utilizes a physiologically based pharmacokinetic (PBPK) model adapted specifically for describing receptor activation throughout the body following compound exposure. A PBPK model is a mechanistic, multi-compartment mathematical model that describes the time course dynamics and overall kinetics of an administered drug dose throughout the organism of interest. PBPK models integrate the physicochemical properties of the substance with the specific physiology of the organism such that the evolution of the ADME (absorption, distribution, metabolism, and excretion) processes can be simulated *in silico*. Drug/substance properties include tissue affinity, membrane permeability, enzymatic stability, etc., whereas the organism/system components include properties such as organ mass/volume and blood flow (Rowland et al., 2011). PBPK modeling is used in this work to couple the pharmacokinetics of a drug to dose-response parameters with the associated off-target receptor in different tissues to generate spatiotemporal dynamics of the off-target receptor activation.

## Results

### Development of the Signaling Pathway Model

As proof of concept, an *in silico* model of the histamine H1 receptor signaling pathway was formulated. This pathway was chosen owing to the well-understood intracellular signaling interactions involved upon receptor stimulation and the existence of a known off-target partial agonist, lisuride (Bakker et al., 2004). The H1 receptor is a G-protein-coupled receptor that, upon activation, leads to dissociation of Gα_q/11_ and the Gβγ complex. Gα_q/11_ activates phospholipase Cβ (PLCβ), leading to hydrolysis of phosphatidylinositol 4,5-biphosphate (PIP_2_) and the formation of inositol triphosphate (IP_3_) and diacylglycerol (DAG) (Bakker et al., 2001, Sandal et al., 2013). IP_3_ mediates a transient intracellular calcium release from the ER (Shah et al., 2015) that eventually mediates the activation of nuclear factor of activated T-cells (NFAT) (Macian, 2005), cAMP response element-binding protein (CREB) (Johannessen and Moens, 2007), and myocyte enhancer factor-2 (Mef2) transcription factors (Lu et al., 2000). DAG simultaneously activates protein kinase C (PKC), and this phosphorylates IκB kinase (IKK), ultimately leading to nuclear factor kappa-light-chain-enhancer of activated B cells (NF-κB) transcription factor activation (La Porta and Comolli, 1997). The Gβγ complex also plays a role in histamine signal transduction, regulating many effectors, including adenylate cyclase (AC) (Maruko et al., 2005) and phosphoinositide 3 kinase (PI3K) (Gautam et al., 1998). AC mediates the subsequent activation of protein kinase A via cyclic AMP (cAMP) leading to CREB phosphorylation and transcription factor activation (Mosenden and Taskén, 2011). PI3K mediates the activation of Akt, NF-κB, and activating transcription factor 2 (ATF2) (Bence et al., 1997, Breitwieser et al., 2007). To provide semi-quantitative information for the relative transcription factor dynamics as described earlier, we assayed pathway perturbations using a luciferase reporter-based transcription factor array to calibrate the fold increase expected of key signaling outputs upon stimulation with an agonist. These transcription factors were identified as NFAT, NF-κB, CREB, Mef2, and ATF2. Incubation of H1 receptor-expressing HeLa cells with histamine showed considerable activation of these transcription factors (Table 1).

**Table 1:** Transcription Factor Changes

Transcription Factor	Fold Change in Relative Luciferase Units
NFAT	1.97 ± 0.063
NF-κB	2.18 ± 1.47
CREB	1.54 ± 0.027
MEF2	2.74 ± 1.31
ATF2	1.67 ± 8.99

Alterations in expression levels of specified genes in the presence of histamine after 6 hr expressed as mean fold changes in relative luciferase units with SD(n = 3) as determined by Cignal reporter assay.

**Figure 1:**
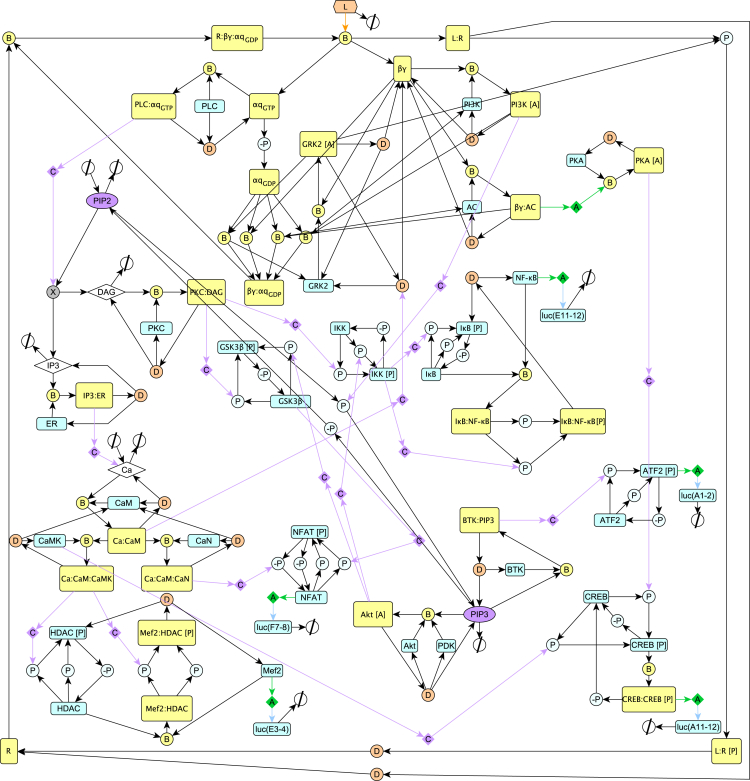
Schematic Representation for the Petri Net of the Histamine H1 Receptor Signaling Pathway Using mEPN Notation The Petri net describes the key relationships between components of the signaling pathway system culminating in the regulation of downstream transcription factor expression stimulated by the binding of a ligand to the histamine H1 receptor.

**Figure 2:**
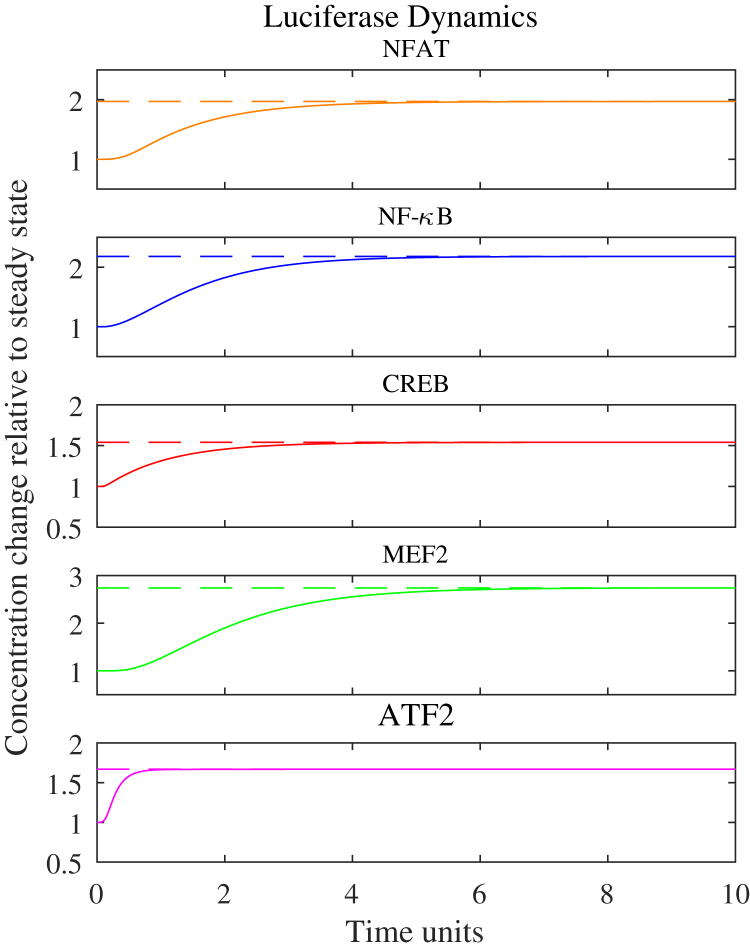
Optimized Transcription Factor Output The ligand (histamine) was introduced at t = 0 (Petri net time units) in the model simulation. Before t = 0 the model was run to steady state. The model solution was fit to the data via optimization of the conserved moieties of the signaling pathway. Dotted lines represent the fold increase in transcriptional activity for the relevant transcription factor observed in the transcription assays. Solid lines represent the normalized model solution for the corresponding transcriptional activity as simulated by luciferase dynamics.

**Figure 3:**
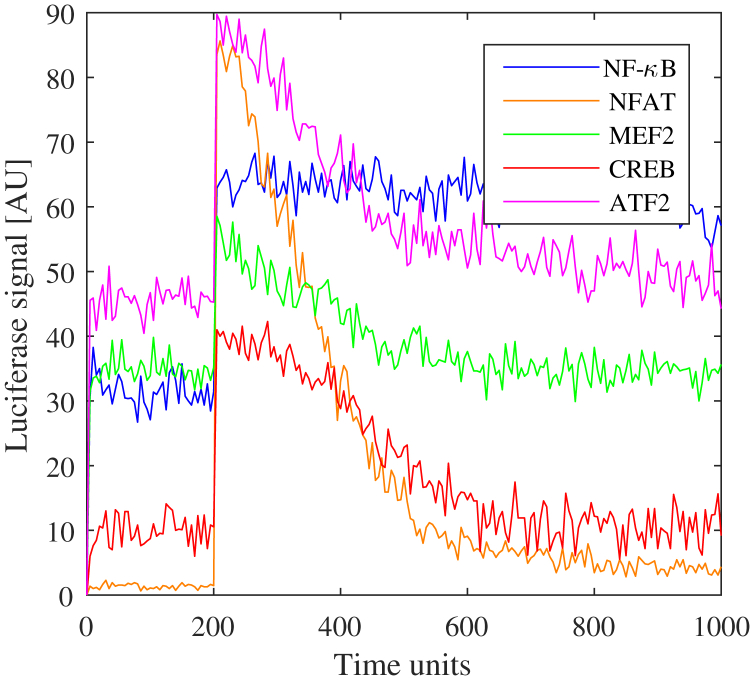
Transient Dynamic Output of the Histamine H1 Receptor Signaling Pathway Using the Stochastic Petri Net This figure illustrates the dynamic output of the stochastic Petri net when a small transient perturbation to the ligand concentration is made at t = 200 units, representing the pre-stimulation steady state. Dynamics are shown for model variables that correspond to luciferase signals for transcription factors associated with a receptor stimulation perturbation.

A stochastic Petri net model of the histamine H1 receptor signaling pathway was formulated based on existing knowledge of the pathway and network interactions with the five critical transcription factors determined to be activated following ligand binding. The pathway in this proof of concept provides an illustrative example of what should ultimately form part of a larger cell signaling model that incorporates the complexity of the known toxicological receptors and associated transcription factors in the proposed methodology. The H1 Petri net includes the key dynamic molecular species and appropriate network interactions that are activated during ligand-binding-induced signaling. This pathway is depicted using the modified Edinburgh pathway notation (mEPN) format (Freeman et al., 2010) in Figure 1 and directly corresponds to the layout of the Petri net. All rates are equal such that all stochastic transitions are equally likely to fire but are effectively modulated by the concentration of upstream reactants in a mass action process. Time is interpreted qualitatively reflecting the relative order of events. Varying quantities in the mathematical model, such as the amount of ligand introduced (“dose”) and the total amounts of system species (i.e., moieties of active and inactive states for each protein), modulate the scale of transcriptional activity regulation, and as such, these values were optimized to correlate with the experimental signaling assays. This optimization was carried out by assuming a large-scale continuum approximation of the Petri net to a system of ordinary differential equations (ODEs) and fitting to the corresponding transcription factor output data (Figure 2). It should be noted that the optimal parameter set is non-identifiable for such a large system with relatively few data points to fit. However, this issue was the precise motivation for the combined Petri net/MCA approach, which is well suited to understanding the relative impact of small perturbations on the transcription factors of interest and prioritize network connectivity information in favor of accurate predictions of parameters and dynamics (Koch et al., 2010). Corresponding pathway reactions, moieties, and ODEs can be found in the supplementary material. In addition to providing static information on the network interactions of the signaling pathway and relative changes in steady state activity following receptor activation, Petri nets can also be used to simulate transient temporal dynamics, providing further dynamic information on the relative order and scale of transcriptional regulation (Figure 3) following a receptor-ligand binding event. However, it is clear that more data would be required for one to relate this dynamic output to the biological context and validate any potential predictions about transient dynamics.

### Analysis of Network Perturbations to Identify Off-Target Responses

**Figure 4:**
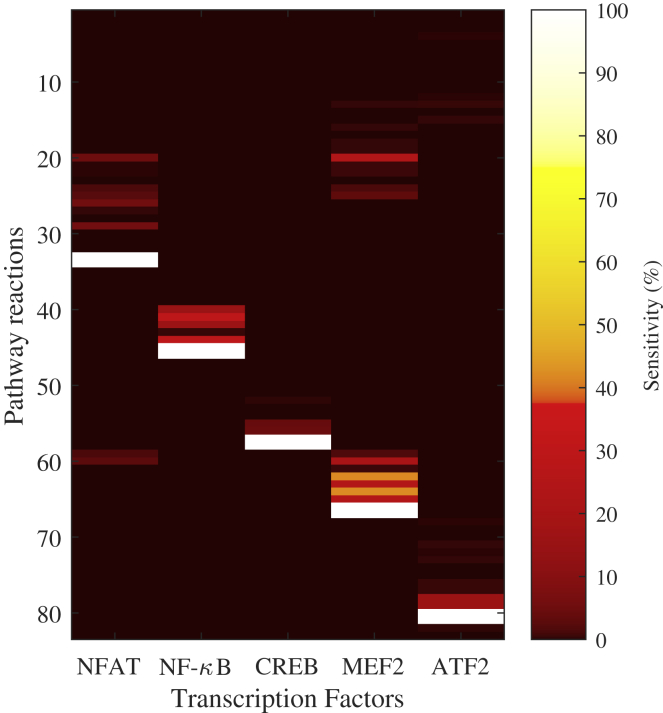
Metabolic Control Analysis (MCA) of the H1 Signaling Pathway Scaled concentration control coefficients as a result of MCA are plotted for the activity of five transcription factors modulated by histamine H1 receptor binding. Each row of the heatmap numerically corresponds to a reaction term in the signaling pathway model (see Supplemental Information). Maximum and minimum values in the heatmap (white patches) represent maximum sensitivity to perturbation of the reaction terms in the model depicting direct transcriptional regulation rates and luciferase decay rates.

The identification of significant pathway reactions upstream of transcription was achieved using metabolic control analysis (MCA), which is a mathematical technique that tests the sensitivity of a given variable to network perturbations (Kacser and Burns, 1973, Heinrich and Rapoport, 1974). Specifically, scaled MCA concentration control coefficients provide the ratio between a relative measure of change in the steady state of a system variable as affected by perturbations in network reaction rates. In our illustrative H1 example model, MCA coefficients were calculated for each transcription factor that was experimentally determined to show significant change in activity following binding of the H1 receptor (Figure 4). The rows of the heatmap in Figure 4 correspond to the numbered reactions as indicated in the supplementary material. MCA points not only to the direct regulation of gene transcription as critical to H1-associated transcriptional activity (white patches in Figure 4), but also to other reactions within the cascade, upstream of the transcription factors and downstream of the target receptor. For example, in this system the transcriptional activity of Mef2 is sensitive to relatively distant biochemical reactions, such as the rate of calcium release from the ER (24% of maximum sensitivity provided by perturbation of Mef2 transcription rate). Also, the model suggests that the transcriptional activity of ATF2 is more sensitive to perturbations in PIP2 synthesis than to the regulation of the BTK:PIP3 complex that directly activates ATF2 by phosphorylation.

The identification of these sensitive perturbation points within the signaling pathway model provide information beyond the transcription factor activity measurements found experimentally, which allows for more optimized, directed experimental designs for receptor identification, if initial screening fails to identify the off-target receptor. For example, for a given compound that was shown to regulate Mef2 transcriptional activity but did not interact with the H1 receptor, this model would inform a proposal to screen for receptors that are known to interact with biochemical reactions identified as being sensitive, such as calcium release, during MCA.

### Translation to Tissue Scales Using a PBPK Model

**Figure 5:**
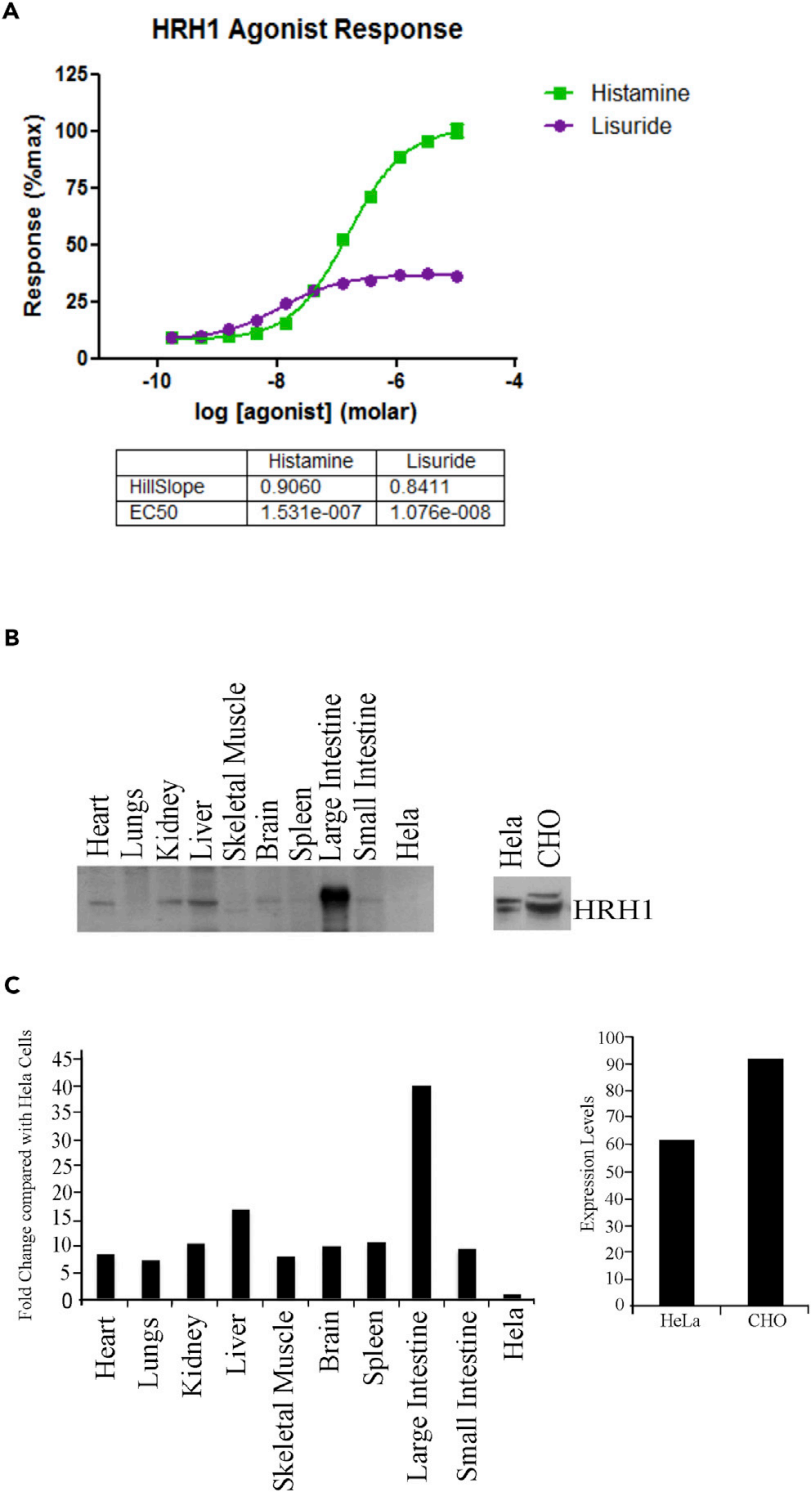
Histamine/Lisuride Dose Response, EC_50_, and Kinetic Parameters (A) Ligand (histamine) and partial agonist (lisuride) dose-response assays used to calculate EC_50_ values. (B) Immunoblotting of H1 receptor in murine organs. (C) Relative quantification of immunoblot relative to HeLa cell lysates.

Following an *in silico* identification of an off-target receptor, extrapolation to the study of potential *in vivo* toxicity can be performed using a PBPK model. For our illustrative example, receptor binding properties are provided by EC_50_ dose-response curves for the off-target H1 agonist, lisuride (Figure 5A), and measurements of the corresponding binding affinity, K_d_ (Bakker et al., 2004). The dose-response curves were estimated by fitting the following equation to the dose-response data:

(Equation 1) $$\mathrm{Response}\%=Min+\frac{\left(Max-Min\right)L^{n}}{EC_{50}^{n}\;+L^{n}}\text{,}$$

for ligand concentration *L*. The optimized parameter values are given in Table 2. To provide tissue-specific responses we also used western blot measurements of relative H1 receptor expression in different tissues (Figures 5B and 5C) and calculated modified tissue-specific EC_50_ values using

$$EC_{50_{i}}=\frac{K_{d}EC_{50}}{R_{i}\left(K_{d}+EC_{50}\right)-EC_{50}}$$

where *i* denotes the *i*^th^ tissue, *K*_*d*_ is the dissociation equilibrium constant for lisuride, and *R*_*i*_ is a measure of receptor abundancy in tissue *i* (see Table 3). For simplicity, this model assumes that the same amount of receptor binding is required to achieve 50% response in each tissue in the absence of any other information, particularly as the response measured is proximal to receptor binding attenuating any potential amplification effects arising from potential signaling cascades in different tissues (Kenakin, 2009). For further information regarding this derivation see the Supplemental Information.

**Table 2:** Kinetic Parameters of Lisuride and the Histamine H1 Receptor

Parameter	Value	SE	Units
*Min*	7.98%	1.066	**/**
*Max*	36.55%	0.5863	**/**
*logEC*_50_	−7.968	0.06724	mol/L
*n* (Hill coefficient)	0.8411	0.1009	**/**
*K*_*d*_	8 × 10^−9^	0.0577	mol/L

Receptor activation of the H1 histamine receptor was studied with known agonist (histamine) and off-target agonist (lisuride). Using these assays, each parameter was calculated using GraphPad Prism.

**Table 3:** Relative Amounts of Histamine H1 Receptor in Murine Tissue Calculated Using Immunoblot Analysis

Parameter	Value	Tissue
*R*_*HE*_	5.60	Heart
*R*_*LU*_	3.56	Lungs
*R*_*KI*_	6.64	Kidney
*R*_*LI*_	11.63	Liver
*R*_*BO*_	3.88	Skeletal muscle
*R*_*BR*_	5.78	Brain
*R*_*SP*_	5.83	Spleen
*R*_*SI*_	5.56	Small intestine
*R*_*CO*_	25.90	Large intestine

Values were used to calculate tissue-specific receptor scaling factors for lisuride EC_50_ values when binding to the histamine H1 receptor.

**Figure 6:**
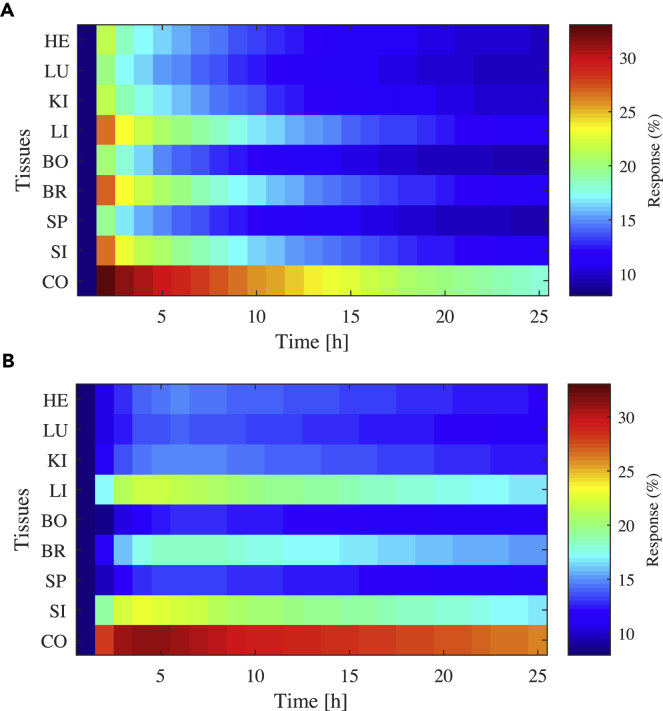
Temporal Tissue Response Predicted by PBPK Modeling Following Doses of Lisuride (A) 25 μg/mL administered intravenously. (B) 0.1 mg administered orally. Tissues are labeled as follows: heart (HE), lungs (LU), kidneys (KI), liver (LI), bone (BO), brain (BR), spleen (SP), small intestine (SI), and colon (CO).

To simulate the pharmacokinetics of lisuride throughout the body, physicochemical properties of the compound were required, which were obtained from previously published measurements. These properties include lipophilicity, whether the drug is neutral/acid/base, solubility (obtained from the DrugBank database [Wishart et al., 2006]), molecular weight (O'Neil, 2013), acid dissociation constant (Meloun et al., 2005), and effective permeability (Winiwarter et al., 1998). The time course dynamics simulated by the PBPK model for drug concentration in each tissue compartment of the body were then coupled to receptor binding properties and relative receptor expression in tissues to provide a predictive temporal response throughout the body. This response can be produced for any dosage regimen and various methods of administration, such as intravenous, oral, and inhalation. The PBPK model was based on the form derived by Peters (2008). The model was optimized for lisuride physicochemical and binding properties and the H1 receptor distribution throughout the different tissues. Example lisuride response kinetics following both intravenous (IV) and oral administrations can be found in Figure 6. The IV dose of 25 μg/mL used in Figure 6 was the same as that used in a previous pharmacokinetic study for relevance (Krause et al., 1991). These experimental data were also the IV data used to optimize the PBPK model to recapitulate the lisuride dynamics in the venous blood compartment and also simulate corresponding oral profiles as per the methodology described by Peters (2008). The oral dose of 0.1 mg chosen for the PBPK model was deemed relevant by matching previous pharmacological studies (Koizumi et al., 1985, Al-Sereiti and Turner, 1989). The dynamic response of the H1 receptor is visualized over time as a solution to Equation (1) with tissue-specific EC_50_ values for the pharmacokinetics of lisuride (*L*) in different parts of the body. Both IV and oral administration simulations are plotted to also highlight the impact of delivery route. This is particularly pertinent in this case where we are studying a receptor that has a relatively high concentration in the gastrointestinal tract. IV administration results in relatively high receptor stimulation in the liver, brain, small intestine, and colon at earlier times, whereas oral administration results in a more gradual accumulation in these tissues and the receptors in the colon are stimulated at a near-maximal level for a relatively long time after oral ingestion. These simulations allow us to compare how the off-target response varies throughout the body over time depending on the pharmacokinetics of the drug coupled with physiologically relevant receptor availability and receptor binding information. Such information is potentially useful to determine whether or not an identified off-target agonist is likely to elicit an off-target receptor response in an area of high target density based on its physicochemical properties.

## Discussion

ADRs are a major cause of patient morbidity, mortality, and drug attrition during development (Pirmohamed et al., 2004). This can be attributed to a poor understanding of the mechanisms underlying the toxic response and also to a lack of current tools for the prediction of a toxic outcome. Animal models have a limited scope, and data obtained using such models may not be ideal for ascertaining toxicity seen in humans. As such, computational systems biology models can be essential tools to improve chemical reaction predictivity (Krewski et al., 2010). In this study, we describe a new *in silico* modeling method that can be used to enhance the current knowledge of pathway perturbations to provide a new toxicity-testing paradigm based on human biology. In this method, chemical-mediated activation of transcription factors and intracellular signaling pathway molecules were used as readouts to inform and drive a pathway-based *in silico* approach to identify possible upstream receptor(s) engaged by such chemicals. *In vitro* data were then used to inform a PBPK *in silico* modeling platform to understand and rank the risk of toxicity at tissue, organ, and whole-body levels over time. Key to this integrative approach was the coupling of *in vitro* experimental techniques and advanced *in silico* modeling to create a unique resource that, with further development and parameterization, could be used to predict the off-target toxicity of compounds that can then inform and direct more focused *in vivo* experimentation.

Mathematical modeling was used to mechanistically describe the processes that lead to regulation of transcriptional activity following the binding of ligand to receptor. This was achieved by designing a signaling pathway model that represented all the relevant processes and biochemical reactions downstream of ligand binding, culminating in the regulation of transcription. We have established a novel *in vitro/in silico* approach using data from assays measuring transcription factor activation and chemically induced perturbations of intracellular signaling pathways to inform *in silico* pathway modeling. This unbiased pathway-led approach uses computational simulations to identify causality between receptor activation and pathway perturbations to aid identification of the upstream receptor/s engaged by the initial MIE. As proof of concept, an *in silico* Petri net model of the histamine H1 receptor-signaling pathway was formulated with the off-target compound, lisuride. The output of this system provides semi-quantitative temporal dynamics for the entire pathway that can be used to investigate system perturbations, simulate experiments, and provide structural pathway predictions. *In vitro* reporter assay data were then used to parameterize and validate the model, and the identification of critical candidate perturbation points was achieved using MCA. Signaling pathway models can be purposely used in this methodology to provide a library of MCA coefficients for a range of transcription factors associated with receptor binding and toxicity and guide further experimentation. In the example shown, calcium release from the ER and PIP2 synthesis are highlighted as important upstream events for the transcriptional activity of Mef2 and ATF2. If a new compound is shown to induce the activity of these transcription factors but the receptor responsible is not identified via screening, for instance, further testing could be guided toward targets that modulate these upstream processes. This illustrates the feasibility of this approach in directing further experimentation toward relevant pathway mechanisms or receptor clusters during the process of receptor identification via focused *in vitro* assay testing.

*In vitro* to *in vivo* extrapolations of whole-body consequences of receptor binding was explored using PBPK modeling. The structure of PBPK models typically revolves around the anatomical structure of the organism, with different organs and tissues of varying perfusion rates being separated into distinct compartments. These compartments are then coupled through the circulation, whose arterial and venous flow is described to connect the organs in a physiological way. Entrance points (e.g., absorption) of the model depend on the drug administration method (e.g., inhalation, ingestion, injection), whereas exit points (e.g., excretion) are generally described via the kidneys and intestine. The flow kinetics of the model determine distribution, whereas metabolism occurs in the liver and intestine. The inherent physiological basis distinguishes true PBPK models from their PK model counterparts that usually simplify the physiology to fewer hypothetical compartments of different flow rates, driven by the data/process of interest, such that they are often more tractable analytically. In contrast, PBPK models are generally more complex but are designed to have a better global representation such that valid extrapolations can be made and disparate experimental data can be integrated during model parameterization. In this way, PBPK models are less reliant on data fitting to obtain appropriate values for equation parameters and essentially the same model (with appropriate modifications) can be suitably applied in many different pharmacological scenarios for quantitative risk assessment and therapy optimization.

PBPK model simulations are increasingly being used in pharmacology, in both academia and industry, to provide important predictions of the pharmacokinetic properties and toxic potential of new drugs at an early stage in drug development (Zhao et al., 2011, Jones and Rowland-Yeo, 2013, Tsamandouras et al., 2015). This type of *in silico* testing can offer a quicker, cheaper, and more ethical alternative method when compared with traditional *in vivo* experiments performed. Ideally, both experimental and computational methods are used harmoniously to provide a cycle of information and enhanced knowledge iteration as the accuracy of PBPK models inevitably rely on quality experimental data to calibrate rates within the differential equations. In the method reported here, physicochemical properties of the chemical are combined with tissue-specific receptor expression and EC_50_ data to predict time course dynamics of the chemical concentrations in each tissue, as well as tissue-level receptor-activation responses to that chemical. These predictions can be produced for any dosage regimen and various methods of administration. In the example study of the off-target partial agonist of the histamine H1 receptor, lisuride, the combination of lisuride pharmacokinetics and relative H1 receptor distribution throughout the body allowed us to predict that the dose response would be most significant in the brain, liver, and gastrointestinal system. In this case example, these results are supported by prior knowledge of the compound and receptor, although the modeling was done agnostic of such prior *in vivo* findings. In particular, receptor response localized to the brain is somewhat expected since lisuride is primarily a psychotherapeutic drug, affecting dopamine and serotonin regulation (Marona-Lewicka et al., 2002). Lisuride is primarily metabolized in the liver, where there is a relatively high expression of histamine receptors. There is also high receptor expression in the gastrointestinal tract owing to the role of histamine in intestinal secretion and motility (Leurs et al., 1995, Sander et al., 2006). Furthermore, lisuride administration in patients with Parkinson disease has been associated with gastrointestinal side effects (Ebadi and Pfeiffer, 2004). Although relative response rates have been quantified by the model in different parts of the body at different times, to translate what such a response directly represents in the context of toxicity and clinical relevance is very complicated, and restricted in this methodology, establishing a challenge beyond the scope of this paper. However, these PBPK-based extrapolations do allow us to generate predictive data relevant to risk assessment and further translation to toxicity at the organ and whole-body levels for off-target receptor perturbations. The output provided by this method is intended to identify toxic potential and guide subsequent *in vitro* and *in vivo* experimentation to organs of interest/importance.

The operating parameters of the approach are circumscribed by the extent of current knowledge regarding receptors and their function. This represents a potential limitation of the strategy, although the mathematically driven signaling pathway model has the potential to identify novel, uncharacterized receptor targets. The challenge of identifying sensitive perturbation points within large-scale networks of receptor signaling pathways required that a semi-quantitative network-based approach be used. This inevitably limits the amount of predictive, dynamic information that can be extrapolated, and caution must be exercised such that the utility of mathematical models is preserved by acknowledging the relevant application that stimulated its design. The approach is experimental (with elements of modeling and extrapolation to assess and rank toxicological risk) and does not incorporate prediction of receptor binding based on chemical or receptor structures. The strength of the methodology is predicated on currently available, validated experimental methods, as it does not require the development of new, untested technologies and relies on sound criteria-based selection of receptors and quantifying receptor function and binding using established experimental techniques. Future work requires the development of multiple pathway models based on training chemical data as well as the integration of pathways, which should be optimized and validated with non-training data. Furthermore, the current PBPK framework can be extended to ensure improved predictive potential by incorporating mechanistic tissue models, catering for a wider range of chemicals and capturing population-level responses. More work is also needed to translate tissue-level receptor activation responses to measures of toxicity, such as relevant biomarkers. Carefully calculated person-to-person variation and covariances within organism-related parameters would also allow for the prediction of a population response whereby different individuals within a sample population may exhibit different levels of exposure and therefore associated toxicity from the same dosage levels. The combined *in vitro/in silico* approach of this study has shown how the multidisciplinary, iterative process of systems biology can be applied to direct experiments, optimize the utility of generated data, and challenge and refine theoretical modeling to improve methods for detecting and predicting toxicity caused by compounds that bind to off-target receptors.

## Methods

All methods can be found in the accompanying Transparent Methods supplemental file.

## Supplemental Information

Document S1. Transparent Methods, Figures S1 and S2, and Tables S1–S4
