# Supplementary material for: A Combined In Vitro/In Silico Approach to Identifying Off-Target Receptor Toxicity
Source: iScience. 2018 May 18;4:84–96. doi: 10.1016/j.isci.2018.05.012 (PMC6147237; doi:10.1016/j.isci.2018.05.012)
Supplement: Document S1. Transparent Methods, Figures S1 and S2, and Tables S1–S4 [file mmc1.pdf]

ISCI, Volume *4*

Supplemental Information

A Combined *In Vitro/In Silico* Approach to Identifying Off-Target Receptor Toxicity

Joseph Leedale, Kieran J. Sharkey, Helen E. Colley, Áine M. Norton, David Peeney, Chantelle L. Mason, Jean G. Sathish, Craig Murdoch, Parveen Sharma, and Steven D. Webb

Supplementary Material

Transparent Methods

Experimental Methods

Cell culture

HeLa cells were cultured in Dulbecco's Modified Eagle Medium supplemented with 10% foetal bovine serum, 2 mM glutamine and incubated at 37 °C in a 5% CO_2 humidified atmosphere.

Quantification of transcription factor activation

Transcription factor activation was measured using Cignal Reporter dual-luciferase assay (Qiagen) as per manufacturer's instructions, to determine intracellular signal transduction perturbation and transcription factor activation data for *in silico* modelling. Briefly, HeLa cells were plated in 96-well plates and transfected with inducible transcription factor responsive constructs composed of specific transcription factor response elements linked to firefly luciferase along with a constitutively expressing *Renilla* construct as an internal control. Transfection was performed with Attractene transfection reagent (Qiagen) as per manufacturer's instructions. Transfected cells were stimulated with histamine (Sigma) for 6 or 24 h and activation of specific transcription factors quantified by luminometer using the Dual-Luciferase Reporter Assay System (Promega).

Receptor activation

Activation of histamine receptor H1 with its known agonist, histamine and the off-target partial agonist, lisuride was determined using PathHunter® Chinese Hamster Overy (CHO)-K1 reporter cells that over-express ProLink™-tagged human histamine H1 receptor and the Enzyme Acceptor (EA)-tagged β-Arrestin (DiscoverX) as per manufacturer's instructions. In these cells activation of histamine receptor H1 by a ligand forces H1 receptor coupling with EA-tagged β-Arrestin leading to the formation of a functional enzyme that is able to generate a chemiluminescent signal upon substrate cleavage. These assays were used to calculate EC50 values for both histamine and lisuride.

Immunoblotting analysis

Mouse organs from wild type C57 mice (Charles River) were snap frozen in liquid nitrogen and whole tissue lysates homogenised in lysis buffer (250 mM sucrose, 50 mM Tris.HCl,1 mM $MgCl_2$, 1% Triton X-100, 1 mM PMSF). Lysates were also generated from HeLa and CHO cells over-expressing histamine H1 receptor. The lysates were centrifuged at 5000 g for 15 minutes at 4 °C and 20 µg of total protein separated using SDS-PAGE and analysed using standard immunoblotting techniques. Membranes were blocked overnight in 5% milk powder in blocking buffer (0.2% Triton X-100 in PBS) at 4 °C and then incubated with rabbit polyclonal anti-H_1R antiserum (Abcam; 1:1000) followed by horseradish peroxidase-conjugated goat anti-rabbit antiserum (Jackson Labs; 1:2500). Immuno-reactive proteins were

visualized using enhanced chemiluminescent substrate detection (ThermoFisher). Densitometry analysis was carried out using Image J and fold-changes in expression relative to HeLa cells calculated.

Petri Nets

The stochastic Petri net model of the histamine H1 receptor signalling pathway was formulated using Snoopy software (Heiner et al., 2012). In the context of cellular signalling pathways, a Petri net is a bipartite graphical representation of a biochemical network with "tokens"/dots-or-numbers (representing number of molecules or concentration) in "places"/circles (network species, proteins etc.). A reaction is represented by the "firing" of a "transition"/square where tokens are moved from one place to a downstream place as indicated by connecting "arcs"/arrows. A transition may fire only if all upstream places contain sufficient tokens, i.e. all reactants must be present for a specific reaction to occur. Petri nets use mass action and Gillespie algorithm principles such that transitions with a higher number of upstream tokens fire with a higher probability.

Parameter Optimisation and Metabolic Control Analysis

The signalling pathway model large-scale continuum approximation to an ODE system was solved using a Runge-Kutta 4/5th order method with Matlab R2015b software. Pathway moieties (conserved quantities defined in supplementary material S1.2) were optimised such that model signalling output was consistent with experimental data. Optimisation was carried out by minimising the error between fold-increase in transcription factor levels in the model and the data. Metabolic scaled concentration control coefficients were calculated using Copasi 4.15 software (Hoops et al., 2006). The Peters (2008) PBPK model code was written and solved with Matlab R2015b software.

1 Modelling the H1 receptor signalling pathway

Table S1, related to Figure 1: Histamine H1 receptor signalling pathway reactions. Each reaction in the Petri net of Figure 1 is described explicitly with associated description and mass action kinetics.

Reaction No.	Reaction	Description	Mass action flux terms
1.	$L + R{:}\beta\gamma{:}\alpha q_{GDP} \rightarrow L{:}R + \beta\gamma + \alpha q_{GTP}$	Ligand binding causes dissociation of αq_{GTP} (i.e. GTP bound αq) and $\beta\gamma$ from the receptor	$v_1 = L * R{:}\beta\gamma{:}\alpha q_{GDP}$
2.	$GRK2 + \beta\gamma \rightarrow GRK2^*$	Activation of G protein coupled receptor kinase 2 ($GRK2$) by binding to free $\beta\gamma$	$v_2 = GRK2 * \beta\gamma$
3.	$GRK2 + \beta\gamma \xleftarrow{CaM:Ca+background} GRK2^*$	$CaM{:}Ca$ (defined below) mediated plus background deactivation of $GRK2$	$v_3 = GRK2^* * CaM{:}Ca + GRK2^*$
4.	$\alpha q_{GTP} \rightarrow \alpha q_{GDP}$	Background hydrolysis of $\alpha q_{GTP}$to αq_{GDP}	$v_4 = \alpha q_{GTP}$
5.	$\alpha q_{GDP} + GRK2^* \rightarrow GRK2 + \beta\gamma{:}\alpha q_{GDP}$	αq_{GDP} will scavenge $\beta\gamma$ from complexes to give a $\beta\gamma{:}\alpha q_{GDP}$ complex	$v_5 = \alpha q_{GDP} * GRK2^*$
6.	$L{:}R \xrightarrow{GRK2^*} pL{:}R$	$GRK2^*$ phosphorylates agonist-activated receptors	$v_6 = L{:}R * GRK2^*$
7.	$pL{:}R \rightarrow R$	The phosphorylated receptors are recognized by arrestins that bind to the receptor which targets it for internalization	$v_7 = pL{:}R$
8.	$L{:}R \rightarrow R$	Background degradation of ligand	$v_8 = L{:}R$

Reaction No.	Reaction	Description	Mass action flux terms
9.	$\beta\gamma + \alpha q_{GDP} \rightarrow \beta\gamma{:}\alpha q_{GDP}$	αq_{GDP} binding with free $\beta\gamma$	$v_9 = \beta\gamma * \alpha q_{GDP}$
10.	$\beta\gamma{:}\alpha q_{GDP} + R \rightarrow R{:}\beta\gamma{:}\alpha q_{GDP}$	Binding of $\beta\gamma{:}\alpha q_{GDP}$ to free receptor to give $R{:}\beta\gamma{:}\alpha q_{GDP}$	$v_{10} = \beta\gamma{:}\alpha q_{GDP} * R$
11.	$\alpha q_{GTP} + PLC \rightarrow PLC{:}\alpha q_{GTP}$	PLC and αq_{GTP} binding	$v_{11} = \alpha q_{GTP} * PLC$
12.	$PLC{:}\alpha q_{GTP} \rightarrow PLC + \alpha q_{GDP}$	Dissociation of $PLC{:}\alpha q_{GTP}$ hydrolyses αq_{GTP} to αq_{GDP}	$v_{12} = PLC{:}\alpha q_{GTP}$
13.	$\emptyset \rightarrow PIP2$	Background production/synthesis	$v_{13} = k_1$
14.	$PIP2 \rightarrow \emptyset$	Background decay/utilisation	$v_{14} = PIP2$
15.	$PIP2 \xrightarrow{PLC:\alpha q_{GTP}} IP3 + DAG$	$PLC{:}\alpha q_{GTP}$ catalysed separation of $PIP2$ into $IP3$ and DAG	$v_{15} = PIP2 * PLC{:}\alpha q_{GTP}$
16.	$IP3 \rightarrow \emptyset$	Background decay/utilisation	$v_{16} = IP3$
17.	$DAG \rightarrow \emptyset$	Background decay/utilisation	$v_{17} = DAG$
18.	$IP3 + ER \rightarrow IP3{:}ER$	$IP3$ binding to receptors on the ER (endoplasmic reticulum)	$v_{18} = IP3 * ER$
19.	$IP3 + ER \leftarrow IP3{:}ER$	$IP3{:}ER$ dissociation	$v_{19} = IP3{:}ER$
20.	$\emptyset \xrightarrow{IP3:ER+background} Ca$	$IP3{:}ER$ induces Calcium (Ca) release from the ER. Plus background release/production	$v_{20} = IP3{:}ER + k_2$
21.	$Ca \rightarrow \emptyset$	Ca is sequestered back in to ER	$v_{21} = Ca$

Reaction No.	Reaction	Description	Mass action flux terms
22.			
23.	$Ca + CaM \rightarrow CaM{:}Ca$	Ca binds with Calmodulin (CaM).	$v_{22} = Ca * CaM$
24.	$Ca + CaM \leftarrow CaM{:}Ca$	$CaM{:}Ca$ dissociation	$v_{23} = CaM{:}Ca$
25.	$CaM{:}Ca + CaN \rightarrow CaM{:}Ca{:}CaN$	$CaM{:}Ca$ binds to Calcineurin (CaN).	$v_{24} = CaM{:}Ca * CaN$
26.	$CaM + CaN \leftarrow CaM{:}Ca{:}CaN$	$CaM{:}Ca{:}CaN$ dissociation	$v_{25} = CaM{:}Ca{:}CaN$
27.	$pNFAT \xrightarrow{CaM:Ca:CaN} NFAT$	$CaM{:}Ca{:}CaN$ induced dephosphorylation of $pNFAT$	$v_{26} = pNFAT * CaM{:}Ca{:}CaN$
28.	$pNFAT \rightarrow NFAT$	(background) dephosphorylation of $pNFAT$	$v_{27} = pNFAT$
29.	$NFAT \xrightarrow{GSK3\beta} pNFAT$	$pNFAT$ = inactive $NFAT$ (no signal)	$v_{28} = GSK3\beta * NFAT$
30.	$NFAT \rightarrow pNFAT$	(background) phosphorylation of $NFAT$	$v_{29} = NFAT$
31.	$GSK3\beta \xrightarrow{PKC:DAG} pGSK3\beta$	$PKC{:}DAG$ catalysed phosphorylation of glycogen synthase kinase 3 beta ($GSK3\beta$)	$v_{30} = GSK3\beta * PKC{:}DAG$
32.	$GSK3\beta \xrightarrow{Akt^*} pGSK3\beta$	Akt^* catalysed phosphorylation of $GSK3\beta$	$v_{31} = GSK3\beta * Akt^*$
33.	$GSK3\beta \leftarrow pGSK3\beta$	Dephosphorylation	$v_{32} = pGSK3\beta$
34.	$\emptyset \xrightarrow{NFAT} luc(\mathrm{F7}-8)$	Luciferase F7-8 signal	$v_{33} = NFAT$
35.	$luc(F7-8) \rightarrow \emptyset$	Decay of luciferase signal	$v_{34} = luc(F7-8)$
36.	$PKC + DAG \rightarrow PKC{:}DAG$	Binding	$v_{35} = PKC * DAG$
37.	$PKC + DAG \leftarrow PKC{:}DAG$	Dissociation	$v_{36} = PKC{:}DAG$

Reaction No.	Reaction	Description	Mass action flux terms
38.			
39.	$IKK \xrightarrow{PKC:DAG} IKKp$	$PKC:DAG$ catalysed phosphorylation of IKK	$v_{37} = IKK * PKC:DAG$
40.	$IKK \xrightarrow{Akt^*} IKKp$	Akt^* catalysed phosphorylation of IKK	$v_{38} = IKK * Akt^*$
41.	$IKK \leftarrow IKKp$	Dephosphorylation	$v_{39} = IKKp$
42.	$I\kappa B \xrightarrow{IKKp+background} pI\kappa B$	$IKKp$ catalysed phosphorylation of $I\kappa B$.	$v_{40} = I\kappa B * IKKp + I\kappa B$
43.	$I\kappa B \leftarrow pI\kappa B$	Dephosphorylation	$v_{41} = pI\kappa B$
44.	$NF\kappa B + I\kappa B \rightarrow I\kappa B: NF\kappa B$	Binding	$v_{42} = NF\kappa B * I\kappa B$
45.	$I\kappa B: NF\kappa B \xrightarrow{IKKp+background} pI\kappa B: NF\kappa B$	$IKKp$ catalysed phosphorylation of $I\kappa B$	$v_{43} = I\kappa B: NF\kappa B * IKKp + I\kappa B: NF\kappa B$
46.	$pI\kappa B: NF\kappa B \rightarrow pI\kappa B + NF\kappa B$	Dissociation	$v_{44} = pI\kappa B: NF\kappa B$
47.	$\emptyset \xrightarrow{NFKB} luc(\mathrm{E11} - 12)$	Luciferase E11-12 signal	$v_{45} = NF\kappa B$
48.	$luc(E11 - 12) \rightarrow \emptyset$	Decay of luciferase signal	$v_{46} = luc(E11 - 12)$
49.	$\beta\gamma + AC \rightarrow \beta\gamma: AC$	Binding of Adenylyl Cyclase (AC, an enzyme) to $\beta\gamma$	$v_{47} = \beta\gamma * AC$
50.	$\beta\gamma + AC \leftarrow \beta\gamma: AC$	Dissociation	$v_{48} = \beta\gamma: AC$
51.	$\alpha q_{GDP} + \beta\gamma: AC \rightarrow AC + \beta\gamma: \alpha q_{GDP}$	αq_{GDP} will scavenge $\beta\gamma$ from complexes to give a $\beta\gamma: \alpha q_{GDP}$ complex	$v_{49} = \alpha q_{GDP} * \beta\gamma: AC$
52.	$PKA \xrightarrow{\beta\gamma:AC} PKA^*$	Activation of Protein Kinase A (PKA)	$v_{50} = PKA * \beta\gamma: AC$

Reaction No.	Reaction	Description	Mass action flux terms
53.			
54.	$PKA \leftarrow PKA^*$	Dissociation	$v_{51} = PKA^*$
55.	$CREB \xrightarrow{PKA^*} pCREB$	PKA^* catalysed phosphorylation of $CREB$	$v_{52} = CREB * PKA^*$
56.	$CREB \xrightarrow{CaM:Ca:CaMK} pCREB$	$CaM:Ca:CaMK$ (defined below) catalysed phosphorylation of $CREB$	$v_{53} = CREB * CaM:Ca:CaMK$
57.	$CREB \leftarrow pCREB$	Dephosphorylation	$v_{54} = pCREB$
58.	$pCREB + pCREB \rightarrow pCREB:pCREB$	Homerdimer formation	$v_{55} = pCREB * pCREB$
59.	$CREB + CREB \leftarrow pCREB:pCREB$	Dissociation	$v_{56} = pCREB:pCREB$
60.	$\emptyset \xrightarrow{pCREB:pCREB} luc(\text{A}11 - 12)$	Luciferase A11-12 signal	$v_{57} = pCREB:pCREB$
61.	$luc(A11 - 12) \rightarrow \emptyset$	Decay of luciferase signal	$v_{58} = luc(A11 - 12)$
62.	$CaM:Ca + CaMK \rightarrow CaM:Ca:CaMK$	$CaM:Ca$ binding reversibly to CaM kinases ($CaMK$).	$v_{59} = CaM:Ca * CaMK$
63.	$CaM + CaMK \leftarrow CaM:Ca:CaMK$	Dissociation	$v_{60} = CaM:Ca:CaMK$
64.	$Mef2:HDAC \xrightarrow{CaM:Ca:CaMK+background} Mef2:pHDAC$	$CaM:Ca:CaMK$ catalysed + background phosphorylation of $HDAC$ within the $Mef2:HDAC$ complex.	$v_{61} = Mef2:HDAC * CaM:Ca:CaMK + Mef2:HDAC$
65.	$Mef2:pHDAC \rightarrow Mef2 + pHDAC$	Dissociation	$v_{62} = Mef2:pHDAC$

Reaction No.	Reaction	Description	Mass action flux terms
66.			
67.	$HDAC \xrightarrow{CaM:Ca:CaMK+background} pHDAC$	$CaM:Ca:CaMK$ catalysed + background phosphorylation of free $HDAC$	$v_{63} = HDAC * CaM:Ca:CaMK + HDAC$
68.	$pHDAC \rightarrow HDAC$	Background dephosphorylation	$v_{64} = pHDAC$
69.	$Mef2 + HDAC \rightarrow Mef2:HDAC$	Binding of myocyte enhancer factor-2 ($Mef2$) to $HDAC$.	$v_{65} = HDAC * Mef2$
70.	$\emptyset \xrightarrow{Mef2} luc(\mathrm{E3}-4)$	Luciferase E3-4 signal	$v_{66} = Mef2$
71.	$luc(E3-4) \rightarrow \emptyset$	Decay of luciferase signal	$v_{67} = luc(E3-4)$
72.	$\beta\gamma + PI3K \rightarrow PI3K^*$	Binding to give active phosphoinositol-3-kinase ($PI3K^*$).	$v_{68} = \beta\gamma * PI3K$
73.	$\beta\gamma + PI3K \leftarrow PI3K^*$	Dissociation	$v_{69} = PI3K^*$
74.	$\alpha q_{GDP} + PI3K^* \rightarrow PI3K + \beta\gamma:\alpha q_{GDP}$	αq_{GDP} will scavenge $\beta\gamma$ from complexes to give a $\beta\gamma:\alpha q_{GDP}$ complex.	$v_{70} = \alpha q_{GDP} * PI3K^*$
75.	$PIP2 \xrightarrow{PI3K^*} PIP3$	$PI3K^*$ catalysed production of inositol lipid $PIP3$ from $PIP2$	$v_{71} = PIP2 * PI3K^*$
76.	$PIP3 \rightarrow PIP2$	Catalysed by PTEN (assumed constant)	$v_{72} = PIP3$
77.	$PIP3 \rightarrow \emptyset$	Background decay/utilisation	$v_{73} = PIP3$
78.	$PIP3 + Akt + PDK \rightarrow Akt^*$	Activation of Akt.	$v_{74} = PIP3 * Akt * PDK$
79.	$PIP3 + Akt + PDK \leftarrow Akt^*$	Dissociation	$v_{75} = Akt^*$

Reaction No.	Reaction	Description	Mass action flux terms
80.			
81.	$BTK + PIP3 \rightarrow BTK{:}PIP3$	Reversible binding of $PIP3$ with Bruton's tyrosine kinase (BTK).	$v_{76} = BTK * PIP3$
82.	$BTK + PIP3 \leftarrow BTK{:}PIP3$	Dissociation	$v_{77} = BTK{:}PIP3$
83.	$ATF2 \xrightarrow{BTK:PIP3+background} pATF2$	$BTK{:}PIP3$ catalysed phosphorylation of activating transcription factor 2 ($ATF2$).	$v_{78} = ATF2 * BTK{:}PIP3 + ATF2$
84.	$ATF2 \leftarrow pATF2$	Dephosphorylation	$v_{79} = pATF2$
85.	$\emptyset \xrightarrow{pATF2} luc(\mathrm{A1}-2)$	Luciferase A1-2 signal	$v_{80} = pATF2$
86.	$luc(A1-2) \rightarrow \emptyset$	Decay of luciferase signal	$v_{81} = luc(A1-2)$
87.	$\emptyset \leftrightarrow L$	Basal degradation of ligand (and basal synthesis during infusion simulations)	$v_{82} = k_3 - L$

Table S2, related to Figure 1: List of pathway moieties. Each equation represents a fixed total amount based on a sum of related variables.

1	$\beta\gamma_{total} = \beta\gamma + R{:}\beta\gamma{:}\alpha q_{GDP} + \beta\gamma{:}\alpha q_{GDP} + \beta\gamma{:}AC + PI3K^{*} + GRK2^{*}$	14	$NF\kappa B_{total} = NF\kappa B + I\kappa B{:}NF\kappa B + pI\kappa B{:}NF\kappa B$
2	$R_{total} = R + L{:}R + pL{:}R + R{:}\beta\gamma{:}\alpha q_{GDP}$	15	$AC_{total} = AC + \beta\gamma{:}AC$
3	$\alpha q_{total} = \alpha q_{GTP} + \alpha q_{GDP} + PLC{:}\alpha q_{GTP} + R{:}\beta\gamma{:}\alpha q_{GDP} + \beta\gamma{:}\alpha q_{GDP} = \beta\gamma_{total}$	16	$PKA_{total} = PKA + PKA^{*}$
4	$GRK2_{total} = GRK2 + GRK2^{*}$	17	$CREB_{total} = CREB + pCREB + 2pCREB{:}pCREB$
5	$PLC_{total} = PLC + PLC{:}\alpha q_{GTP}$	18	$CaMK_{total} = CaMK + CaM{:}Ca{:}CaMK$
6	$ER_{total} = ER + IP3{:}ER$	19	$HDAC_{total} = HDAC + pHDAC + Mef2{:}HDAC + Mef2{:}pHDAC$
7	$CaM_{total} = CaM + CaM{:}Ca + CaM{:}Ca{:}CN + CaM{:}Ca{:}CaMK$	20	$Mef2_{total} = Mef2 + Mef2{:}HDAC + Mef2{:}pHDAC$
8	$CN_{total} = CN + CaM{:}Ca{:}CN$	21	$PI3K_{total} = PI3K + PI3K^{*}$
9	$NFAT_{total} = NFAT + pNFAT$	22	$Akt_{total} = Akt + Akt^{*}$
10	$GSK3\beta_{total} = GSK3\beta + pGSK3\beta$	23	$PDK_{total} = PDK + Akt^{*}$
11	$PKC_{total} = PKC + PKC{:}DAG$	24	$BTK_{total} = BTK + BTK{:}PIP3$
12	$IKK_{total} = IKK + IKKp$	25	$ATF2_{total} = ATF2 + pATF2$
13	$I\kappa B_{total} = I\kappa B + pI\kappa B + I\kappa B{:}NF\kappa B + pI\kappa B{:}NF\kappa B$		

Table S3, related to Figure 2: Pathway model ODEs (large-scale continuum approximation). The systems of ODEs representing the histamine H1 receptor signaling pathway are provided below. $d[i]/dt$ represents the rate of change of variable i over time, t. Note that reaction $v(j)$ corresponds to reaction j in Table S1.

Ordinary Differential Equations
$\frac{d[L]}{dt} = v(82) - v(1)$
$\frac{d[\beta\gamma]}{dt} = v(1) + v(3) + v(48) + v(69) - v(2) - v(9) - v(47) - v(68)$
$\frac{d[L:R]}{dt} = v(1) - v(6) - v(8)$
$\frac{d[\beta\gamma:\alpha q_{GDP}]}{dt} = v(5) + v(9) + v(49) + v(70) - v(10)$
$\frac{d[GRK2^*]}{dt} = v(2) - v(3) - v(5)$
$\frac{d[\alpha q_{GDP}]}{dt} = v(4) + v(12) - v(5) - v(9) - v(49) - v(70)$
$\frac{d[pL:R]}{dt} = v(6) - v(7)$
$\frac{d[\alpha q_{GTP}]}{dt} = v(1) - v(4) - v(11)$
$\frac{d[PIP2]}{dt} = v(13) + v(72) - v(14) - v(15) - v(71)$
$\frac{d[IP3]}{dt} = v(15) + v(19) - v(16) - v(18)$
$\frac{d[DAG]}{dt} = v(15) + v(36) - v(17) - v(35)$
$\frac{d[IP3:ER]}{dt} = v(18) - v(19)$
$\frac{d[Ca]}{dt} = v(20) + v(23) - v(21) - v(22)$
$\frac{d[CaM:Ca]}{dt} = v(22) - v(23) - v(24) - v(59)$
$\frac{d[CaM:Ca:CaMK]}{dt} = v(59) - v(60)$

Ordinary Differential Equations
$\frac{d[CaM:Ca:CN]}{dt} = v(24) - v(25)$
$\frac{d[pNFAT]}{dt} = v(28) + v(29) - v(26) - v(27)$
$\frac{d[pGSK3\beta]}{dt} = v(30) + v(31) - v(32)$
$\frac{d[luc(F7\_8)]}{dt} = v(33) - v(34)$
$\frac{d[PKC:DAG]}{dt} = v(35) - v(36)$
$\frac{d[IKKp]}{dt} = v(37) + v(38) - v(39)$
$\frac{d[I\kappa B:NF\kappa B]}{dt} = v(42) - v(43)$
$\frac{d[pI\kappa B:NF\kappa B]}{dt} = v(43) - v(44)$
$\frac{d[pI\kappa B]}{dt} = v(40) + v(44) - v(41)$
$\frac{d[luc(E11\_12)]}{dt} = v(45) - v(46)$
$\frac{d[\beta\gamma:AC]}{dt} = v(47) - v(48) - v(49)$
$\frac{d[PKA^*]}{dt} = v(50) - v(51)$
$\frac{d[pCREB]}{dt} = v(52) + v(53) - v(54) - 2 * v(55)$
$\frac{d[pCREB:pCREB]}{dt} = v(55) - v(56)$
$\frac{d[luc(A11\_12)]}{dt} = v(57) - v(58)$
$\frac{d[pHDAC]}{dt} = v(62) + v(63) - v(64)$
$\frac{d[Mef2:HDAC]}{dt} = v(65) - v(61)$
$\frac{d[Mef2p:HDAC]}{dt} = v(61) - v(62)$

Ordinary Differential Equations
$\frac{d[luc(E3\_4)]}{dt} = v(66) - v(67)$
$\frac{d[PI3K^*]}{dt} = v(68) - v(69) - v(70)$
$\frac{d[PIP3]}{dt} = v(71) + v(75) + v(77) - v(72) - v(73) - v(74) - v(76)$
$\frac{d[Akt^*]}{dt} = v(74) - v(75)$
$\frac{d[BTK:PIP3]}{dt} = v(76) - v(77)$
$\frac{d[pATF2]}{dt} = v(78) - v(79)$
$\frac{d[luc(A1\_2)]}{dt} = v(80) - v(81)$

2 Tissue-specific dose-response relationship derivation

Lisuride response in CHO cells can be described as a function of lisuride concentration (L [mol/L]) as shown in equation (S1) for the parameters given in Table S4 based on data obtained by DiscoverX dose response assay (Figure S1).

$$Response\% = Min + \frac{(Max - Min)L^n}{EC_{50}^n + L^n} \tag{S1}$$

Table S4, related to Figure 6: Agonist response parameters. Parameter values used to describe the dose-response curve in Figure S1.

Parameter	Description	Value	Units
Min	Minimum response	7.98 %	/
Max	Maximum response	36.55 %	/
EC_{50}	Effective concentration	1.076×10^{-8}	mol/L
n	Hill coefficient	0.8411	/

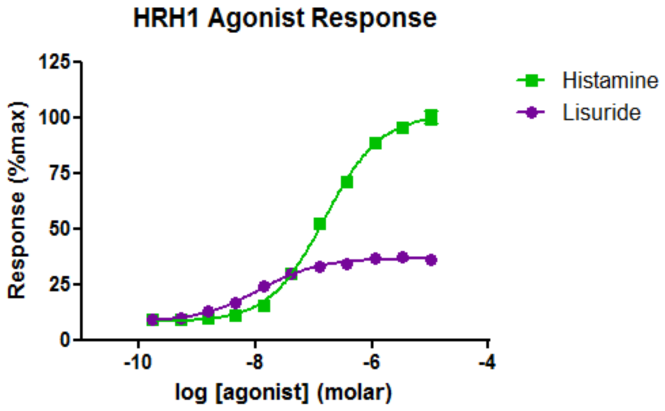

Figure S1, related to Figure 6: Ligand and agonist dose response assay. Dose response assays (DiscoverX) were used to calculate relevant EC_{50} values for both ligand (histamine) and agonist (lisuride).

2.1 *Defining the EC_{50} values for different cells and tissues*

We can describe the number of bound receptors using the equilibrium Langmuir-Hill equation:

$$R_{bound} = R_{total} \frac{L}{K_D + L}$$

where R_{total} represents the total number of receptors (bound + unbound), L represents ligand concentration and K_D is the dissociation equilibrium value.

In CHO cells, let $R_{total} = R_{total}^{CHO}$. For the ligand concentration that causes 50% maximal response (i.e. $L = EC_{50}^{CHO}$) we can derive the corresponding amount of bound receptors, $R_{bound_{50}}^{CHO}$, namely:

$$R_{bound_{50}}^{CHO} = R_{total}^{CHO} \frac{EC_{50}^{CHO}}{K_D + EC_{50}^{CHO}}.$$

We would expect the EC_{50} value to vary between tissues, being dependent on the relative amount of receptors in that tissue. But for simplicity we will assume that the amount of bound receptors required for a half-maximal response is the same, i.e. $R_{bound_{50}}^{CHO} = R_{bound_{50}}^{i}$(for tissue i). Therefore, for tissue i we have a total number of receptors, R_{total}^{i}, and a new EC_{50} value, EC_{50}^{i} defined as below:

$$R_{bound_{50}}^{i} = R_{total}^{i} \frac{EC_{50}^{i}}{K_D + EC_{50}^{i}} = R_{total}^{CHO} \frac{EC_{50}^{CHO}}{K_D + EC_{50}^{CHO}},$$

$$\Rightarrow EC_{50}^{i} = \frac{K_D EC_{50}^{CHO}}{R_{total}^{i/CHO}(K_D + EC_{50}^{CHO}) - EC_{50}^{CHO}},$$

where $R_{total}^{i/CHO} = R_{total}^{i}/R_{total}^{CHO}$. EC_{50}^{CHO} is equal to the EC_{50} found in Table S4 and using the measured K_D for lisuride, we can modify our EC50 value for each tissue and update our dose response function in equation (S1) by measuring the relative amount of receptors in tissue i compared to CHO cells, $R_{total}^{i/CHO}$.

3 Identifiability Analysis

Pathway moieties were optimised to fit the steady state fold-changes of the transcription factors (Figure 2 of the main manuscript). Due to the nature of the approach taken (maximising network/pathway connectivity information rather than focusing on dynamics with a minimal model), there is an inevitable issue with model parameterisation. We have made efforts to find the global optimum for the parameter set through Latin hypercube sampling, but it is clear that other parameterisations could give fits that would at least look as good (by eye) and fit well within any expected variation from the biology/experimental error.

A profile likelihood estimation method was employed to determine parameter identifiability analysis and, as expected, the results indicate that the parameters are not uniquely identifiable for such a problem since the number of free parameters far outnumber the number of data. The analysis was performed using Data2Dynamics software (Raue et al., 2013, Raue et al., 2015) and the profile likelihood method to analyses parameter identifiability, as developed by Prof Jens Timmer's group in Freiburg (Raue et al., 2009, Maiwald et al., 2016). The results of this identifiability analysis are illustrated in Figure S2.

Additionally, local sensitivity analysis indicates that, in line with the complementary MCA method (as described in the main manuscript), it is the nodes that are relatively close to the transcription factor signal that dominate the subsequent expression following receptor activation. These fairly intuitive results suggest that errors arising due to parameter changes throughout the system can be mitigated for in order to achieve the same fold-changes seen experimentally, provided that (a) other parameters are also changed (in the case of sensitive proximal nodes) or (b) because their values are not having a large effect on the downstream signal (distal nodes). This emphasises the importance of the use of MCA in the proposed methodology as a means of identifying any distal nodes that do affect transcription factor signal, as identified in the paper. Of course, to acquire identifiable parameters, far more data would be needed (than is available for such a complex network) or typically, a minimal ODE model would be constructed that significantly reduces the number of variables and parameters that cannot be identified with given data. This minimal approach would potentially be useful for answering questions about the dynamics of the H1-histamine signalling pathway for a very specific scenario and/or ligand. However, this approach would be limited when trying to identify off-target receptor toxicity.

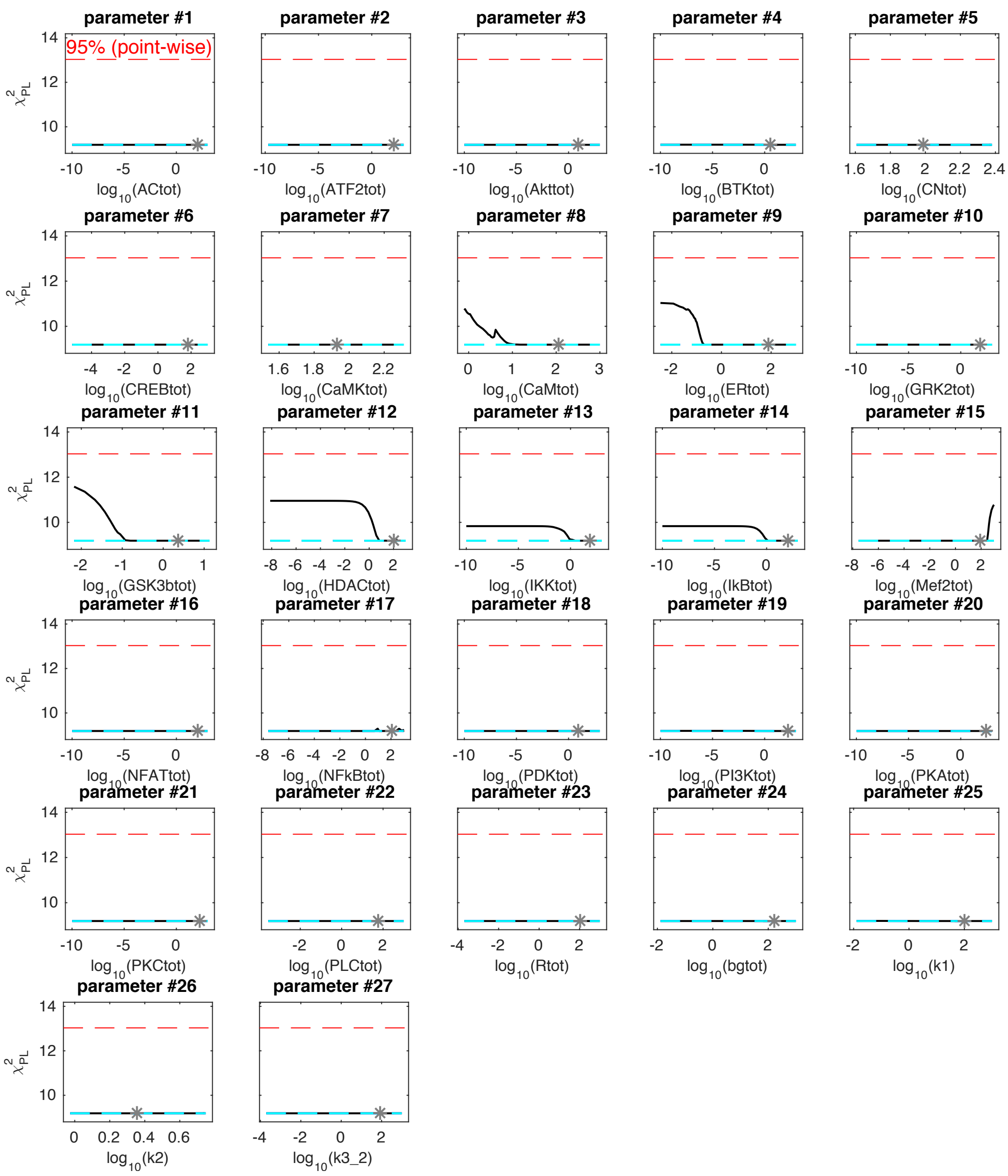

Figure S2, related to Figure 2: Identifiability analysis for the H1 Histamine pathway Petri net. Briefly, following the identification of a globally optimal parameter set each model parameter is perturbed within a prescribed range, one parameter at a time. Starting from the optimal value, a single parameter is perturbed, set to be fixed at the new and non-optimal value, and the model is then re-fit, i.e. by optimising all other parameters. Then corresponding likelihood values are plotted for this new fit. Analysis was conducted using the profile likelihood method in Data2Dynamics software, solved in MATLAB 2017b.

References

Heiner M, Herajy M, Liu F, Rohr C and Schwarick M (2012). Snoopy–a unifying Petri net tool. Application and Theory of Petri Nets, Springer**:** 398-407.

Hoops S, Sahle S, Gauges R, Lee C, Pahle J, Simus N, Singhal M, Xu L, Mendes P and Kummer U (2006) COPASI—a complex pathway simulator. *Bioinformatics* 22: 3067-3074.

Maiwald T, Hass H, Steiert B, Vanlier J, Engesser R, Raue A, Kipkeew F, Bock H H, Kaschek D, Kreutz C and Timmer J (2016) Driving the model to its limit: profile likelihood based model reduction. *PloS one* 11: e0162366.

Peters S A (2008) Evaluation of a generic physiologically based pharmacokinetic model for lineshape analysis. *Clinical pharmacokinetics* 47: 261-275.

Raue A, Kreutz C, Maiwald T, Bachmann J, Schilling M, Klingmüller U and Timmer J (2009) Structural and practical identifiability analysis of partially observed dynamical models by exploiting the profile likelihood. *Bioinformatics* 25: 1923-1929.

Raue A, Schilling M, Bachmann J, Matteson A, Schelke M, Kaschek D, Hug S, Kreutz C, Harms B D, Theis F J, Klingmuller U and Timmer J (2013) Lessons learned from quantitative dynamical modeling in systems biology. *PloS one* 8: e74335.

Raue A, Steiert B, Schelker M, Kreutz C, Maiwald T, Hass H, Vanlier J, Tönsing C, Adlung L, Engesser R, Mader W, Heinemann T, Hasenauer J, Schilling M, Hofer T, Klipp E, Theis F, Klingmüller U, Schoberl B and Timmer J (2015) Data2Dynamics: a modeling environment tailored to parameter estimation in dynamical systems. *Bioinformatics* 31: 3558-3560.
